# Predation Risk Effects of *Harmonia axyridis* on the Development and Fecundity of *Periphyllus koelreuteriae*

**DOI:** 10.3390/insects16070695

**Published:** 2025-07-06

**Authors:** Haibo Yang, Jiaoyi Du, Lei Wang, Pinhong Zhu, Dingxu Li, Jianrong Huang, Zhenjie Hu

**Affiliations:** 1 College of Horticulture and Plant Protection, Henan Provincial Engineering Technology Research Center of Green Plant Protection, Henan University of Science and Technology, Luoyang 471000, China; 18336305071@163.com (J.D.); wlhaust@163.com (L.W.); phzhu@haust.edu.cn (P.Z.); dingxli@haust.edu.cn (D.L.); 2 Institute of Plant Protection, Henan Academy of Agricultural Sciences, Zhengzhou 450002, China; hjrsxx@163.com

**Keywords:** predator–prey interaction, non–consumptive effect, developmental stage–dependent, predation cues, laboratory experiments

## Abstract

In the scenario that requires the biological control of pests using predatory enemies, predators not only prey on pests directly but also can affect the population fitness of pests through indirect non-consumptive effects (predation risk effects). However, the impact of predation risk effects varies depending on the mode of stress imposed by natural enemies and the state of the stressed pests. Herein, we exposed aphids (*Periphyllus koelreuteriae*) at different stages to various cues from the multicolored Asian lady beetle (*Harmonia axyridis*) to assess the effects of different predation risks on *P. koelreuteriae* development and reproduction. We found that the effect of predation risk on aphid developmental time was clearly stage-dependent. When 1st-instar nymphs were exposed to predator cues, their developmental time was prolonged only in the early stages (1st–3rd instar nymphs). Similarly, when third-instar nymphs were stressed, only the current stage (third instar) showed a developmental delay, while fourth-instar nymphs and adult aphids were not significantly affected. Additionally, aphids at different stages perceive predation risk differently. Nymphs cannot recognize visual cues from predators, but can perceive odor cues. In contrast, adult aphids are sensitive to a variety of predator cues, including visual ones. Predation risk caused aphids to shorten their adult lifespan and reduce reproductive output, but it increased the proportion of diapause offspring. This study highlights the importance of considering how predation risk affects multiple life stages and physiological changes, which are important for gaining insights into the mechanisms of predator–prey interactions and for comprehensively assessing the ability of lady beetles to regulate aphid populations.

## Simple Summary

*Periphyllus koelreuteriae* is an important pest of the landscape tree *Koelreuteria bipinnata*, and the multicolored Asian lady beetle (*Harmonia axyridis*) is a common predatory natural enemy of this pest. Understanding the effects of predation risk from *H. axyridis* on the development and reproduction of *P. koelreuteriae* at different developmental stages can contribute to a more comprehensive assessment of the ability of lady beetles to regulate aphid populations. Our study found that nymph aphids could not see the threat of lady beetles but could sense their odor and tactile cues, while adult aphids could detect the visual, odor, and tactile cues of lady beetles. Nymph aphids sensing lady beetle cues only delayed their current developmental stage and did not affect later development or adult longevity, but exhibited reduced fecundity. Adult aphids at risk of predation, on the other hand, had shorter lifespans, a lower reproductive output, and an increased proportion of diapause offspring. The findings strengthen our understanding of predator–prey interactions and contribute to a comprehensive evaluation of the ability of lady beetles to control aphid populations.

## 1. Introduction

One of the most important interspecific interactions in ecology is the constant struggle between a predator and its prey. Predator–prey interactions can be divided into two types: consumptive and non-consumptive effects [1,2]. Predators use consumptive effects to reduce prey densities through lethal predation and can also affect prey populations by influencing prey fitness through non-consumptive effects or predation risk [3,4]. While predation risk does not directly kill prey, it can have adverse effects. Upon sensing a predation threat, prey alter their behavior and physiology, which may decrease their suitability for survival or reproduction [5]. The research indicates that the non-consumptive impacts of predators on their prey can occasionally be more significant than the consumptive impacts [6]. Identifying how prey sense predators and the trait modifications that lead to declines in prey numbers is vital for a thorough understanding of predator effects on prey.

Prey use a range of stimuli, such as visual, auditory, olfactory, and tactile cues, to evaluate the risk of predation in their environment [7,8]. Prey can recognize potential threats and initiate avoidance actions based on visual cues, like predator appearance, body shape, color, and movement patterns [9,10]. Alternatively, prey can assess the threat situation based on specific vocalizations of predators [11]. Volatile chemical cues have been reported to be particularly important in prey assessment of predation risk [12]. However, prey must rely more on visual or tactile evaluations to determine predation risk when olfactory cues are unclear or absent [13]. To understand the impact of non-consumptive effects on prey populations, it is critical to identify mechanisms for detecting predation risk.

When animals correctly evaluate predation risk, they can exhibit effective anti-predator responses. This defensive strategy usually leads to changes in the prey’s behavioral responses, developmental progress, morphological characteristics, physiological traits, and resource allocation [14,15,16]. For example, when there is a threat of predators, the larvae of the potato beetle, *Leptinotarsa decemlineata*, decrease their feeding activity [12]. Under the threat of predation by turtle lady beetles (*Propylea japonica*), *Drosophila melanogaster* exhibits different levels of accelerated development [17]. Offspring of *Daphnia cucullata* have longer helmets in the presence of predator cues, resulting in better survival rates [18]. Exposure to predators of potato beetles increases the risk of being killed by entomopathogenic nematodes and fungi [19]. When female damselflies (*Ischnura cervula*) detect predator signals in the water, they decrease their feeding drive and focus more on locating safe habitats [20].

*Periphyllus koelreuteriae* (Takahashi) is one of the most damaging pests of *Koelreuteria bipinnata* (Franch.) in East Asia [21]. It mainly feeds on the tender shoots, leaves, and other young parts of the tree, causing the leaves to curl up, in severe cases, even leading to the death of branches and leaves. At the same time, the aphid also secretes large quantities of honeydew, which induces the host leaves and lower shrubs to develop sooty mold disease, reducing the ornamental value and even polluting pedestrians [22]. In China, this aphid has two damage peaks each year (in spring and autumn), with both long-winged and short-winged morphs in each generation, but the short-winged morph predominates (about 70%). It overwinters as fertilized eggs in the bark crevices and at the tree forks [23]. The eggs hatch in the early spring of the following year and continue to infest *K. bipinnata* from March to May. Diapausing aphids begin to appear in the field in late April, and the diapause first-instar nymphs oversummer from mid-May to late September, forming a second peak of infestation in late autumn (early October to early November). They overwinter as fertilized eggs from mid-November to mid-March of the following year [23]. Currently, chemical control is still the main control measure for this aphid, and long-term use not only accelerates the development of pesticide resistance but also causes environmental pollution and endangers human health. Biological control is a promising alternative for controlling *P. koelreuteriae*. The multicolored Asian lady beetle, *Harmonia axyridis* (Pallas), is an impressive consumer of aphids, abundant and ubiquitous in agroforestry ecosystems, and is often used as a biological agent to suppress aphid populations [24,25]. However, the predation risk posed by *H. axyridis* on *P. koelreuteriae* has not been reported. In order to improve the effectiveness of biological control, it is necessary to understand the effect of predation risk on *P. koelreuteriae* to maximize the role of lady beetles in aphid control.

Here, we investigated the effect of predation risk from *H. axyridis* on the development and fecundity of different stages (first-instar nymphs, third-instar nymphs, and adults) of *P. koelreuteriae*, including the production of diapause offspring. Four types of predation risks were established: predator visual (PV) cues, predator odor (PO) cues, predator visual and odor (PV + PO) cues, and predator visual, odor, and tactile (PV + PO + PT) cues. The results reveal the detection mechanism of *P. koelreuteriae* in response to the predation risk from *H. axyridis*. This will help evaluate the population control ability of lady beetles over aphids more comprehensively and provide new ideas for environmentally friendly aphid control.

## 2. Materials and Methods

### 2.1. Experimental Insects

Eggs of *P. koelreuteriae* were collected from *K. bipinnata* trees at Henan University of Science and Technology (34°62′ N, 112°42′ E) Luoyang, China, in November 2022. Barks with aphid eggs were taken back to the laboratory and stored at 0 °C until the end of February 2023. Small pieces of bark with eggs were placed in Petri dishes (d = 90 mm) covered with moistened filter paper at the bottom, and then the Petri dishes were placed in an artificial climate chamber (PQX-450B-30H, Ningbo Laifu Technology Co., Ltd., Ningbo, China). The control conditions were set to a temperature of 23 ± 1 °C, a relative humidity of 65% ± 5%, and a photoperiod of 15:9 h (L:D). Hatched nymphs were collected daily at 9:00 and 21:00. The nymphs were reared on branches of *K. bipinnata* that were grown hydroponically. The tested aphids included three groups: first-instar nymphs, third-instar nymphs, and adults.

*H. axyridis* adults were collected from oilseed rape (*Brassica napus*) fields near the campus in early April. They were fed *P. koelreuteriae*, *Myzus persicae*, and *Cinara tujafilina* in a rearing box (13.7 cm × 8.2 cm × 5.3 cm; room temperature), with sufficient aphids provided every day. Efforts were made to ensure that the lady beetles could feed on different species of aphids to maintain their feeding habits and vitality in the wild. Only *H. axyridis* adults were used in the experiment, but without age standardization.

### 2.2. The Effect of H. axyridis Risk on the Development and Reproduction of P. koelreuteriae

**Figure 1:**
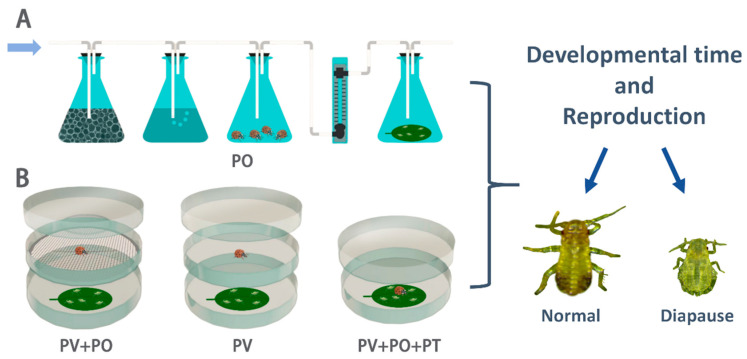
Experimental apparatus used to detect aphid response to predator cues. (**A**) Aphid response to predator odor. (**B**) Aphid responses to predator visual, odor, and tactile cues. PO: predator odor cues; PV: predator visual cues; PV + PO: predator visual and odor cues; PV + PO + PT: predator visual, odor, and tactile cues.

The test apparatus used to detect aphid response to different predator cues was modified according to Hermann et al. (2021) [26]. The effect of lady beetle odors on aphid development and reproduction was assessed using an odor-source test system (Figure 1A). The system consisted of four triangular flasks (50 mL) connected in sequence. First, air flowed through the first triangular flask containing activated charcoal; then, it was humidified in the second triangular flask containing distilled water. The air was then pumped into the third triangular flask containing lady beetles and finally flowed into the last triangular flask containing aphids reared on the leaves. A flow meter controlled the airflow rate at 1.0 L/min. Black cardboard was placed between the two flasks containing lady beetles and aphids to ensure that the aphids could not see the lady beetles and could only sense the volatile odors emitted by them. Prior to each test, one healthy adult lady beetle was randomly selected to be placed in the odor source flask and starved for 12 h in advance. Ten aphids were placed in the triangular flask to be exposed to lady beetle odors for 12 h.

The effects of PV, PV + PO, and PV + PO + PT on aphids were investigated using a modified double-deck Petri dish system (Figure 1B). Predator visual cues test device: consisted of upper and lower Petri dishes. The upper Petri dish contained lady beetles, while the lower Petri dish held aphids. The edges of both dishes were sealed with sealing film. Aphids could see the lady beetles in the upper dish through the glass, but could not detect the volatile odors of the lady beetles. Predator visual and odor cues test device: similarly, it consisted of upper and lower Petri dishes, except that the aphids and lady beetles were separated from each other by a metal mesh between the two dishes, allowing the aphids in the lower Petri dish to perceive predator visual and odor cues simultaneously. Predator visual, odor, and tactile cues test device: aphids and lady beetles were placed together in one Petri dish. Before the test, the mandibles of lady beetles were cut off with a surgical blade, rendering them incapable of feeding on the aphids but not affecting their ability to move around and still have access to the aphids. Meanwhile, aphids were reared individually in separate Petri dishes without lady beetles as a control.

Before each experiment, adult lady beetles were starved for 12 h in advance. One lady beetle was placed as the predation risk source for each treatment group. A total of 10 freshly born nymphs (<6 h old, 1st-instar nymphs) or 3rd-instar nymphs were exposed to predation risk for 12 h. In contrast, adult aphids (<6 h after eclosion) were only treated for 6 h to avoid the effects of adult aphid reproduction. For the control group, 10 freshly born nymphs were also placed in a Petri dish without lady beetles for 12 h. After exposure to each predation risk, aphids were individually fed in Petri dishes with host leaves; the bottoms of the dishes were moisturized with 0.6% agar, and fresh leaves were replaced daily. The development and survival of each individual were recorded at 12 h intervals, and reproduction (the number of nymphs in the first 6 days) and adult longevity were recorded after adult emergence. The number of diapause nymphs in the offspring was also recorded. There were a total of 50 initial aphids per treatment. Each treatment was kept at 23 ± 1 °C, 65% ± 5% relative humidity, and a 15:9 h (L:D) photoperiod.

### 2.3. Statistics

Percentage data (proportion of diapause offspring) were arcsine square root-transformed. Data normality was checked with the Shapiro–Wilk test, and variance homogeneity was assessed using Levene’s test. The effects of predator cues on developmental time, total fecundity, and the proportion of diapause offspring were analyzed by one-way ANOVA with post hoc Turkey’s HSD tests. The reproductive traits were also analyzed using two-way ANOVA with stressed aphid stage and stress type as factors. All statistical analyses were conducted using SPSS Statistics 25 software.

## 3. Results

### 3.1. The Effects of Different Predation Risks on the Development of Aphids

When first-instar nymphs were exposed to different predator cues, the developmental time was significantly prolonged only during the early stages (1–3) (Table 1). There were significant effects on the developmental duration of the first-instar nymphs (N1), second-instar nymphs (N2), third-instar nymphs (N3), and total nymph stages after the exposure of first-instar nymphs to different predation risks (N1: F_4,105_ = 20.904, *p* < 0.001; N2: F_4,105_ = 8.072, *p* < 0.001; N3: F_4,105_ = 4.611, *p* = 0.002; total nymph stage: F_4,105_ = 24.343, *p* < 0.001). However, the developmental duration of fourth-instar nymphs (N4) and adult longevity was not significantly affected (N4: F_4,105_ = 1.101, *p* = 0.360; adult: F_4,105_ = 1.956, *p* = 0.107). In particular, the developmental durations of N1, N2, and N3 in the PV + PO + PT group were significantly longer than those in the control and PV groups, but there was no difference between the PV group and the control group. In addition, there was no significant difference in the developmental durations between the PO group and the PV + PO group.

When the third-instar nymphs were exposed to different predation cues, only the developmental duration of the third-instar nymphs showed significant differences (F_4,85_ = 7.518, *p* < 0.001), but there was no effect on the fourth-instar nymphs and adults (N4: F_4,85_ = 0.290, *p* = 0.883; Adult: F_4,85_ = 1.447, *p* = 0.216). The developmental duration of the third-instar nymphs in the PV + PO + PT group was significantly longer than those in the control and PV groups, but not significantly different from that of the PO and PV + PO groups (Table 2).

When adult aphids were exposed to different predation cues, all four predation risk treatment groups of lady beetles significantly shortened the lifespan of adult aphids (F_4,85_ = 8.578, *p* < 0.001). The lifespan of adult aphids in the PV + PO + PT group was significantly shorter than that in the PV and PO groups, but not significantly different from that in the PV + PO group. Although adult longevity in the PO group was not significantly different from that in the PV group, it was significantly shorter in the PV + PO group than in the PV group (Table 3).

### 3.2. The Effects of Different Predation Risks on the Reproduction of Aphids

**Figure 2:**
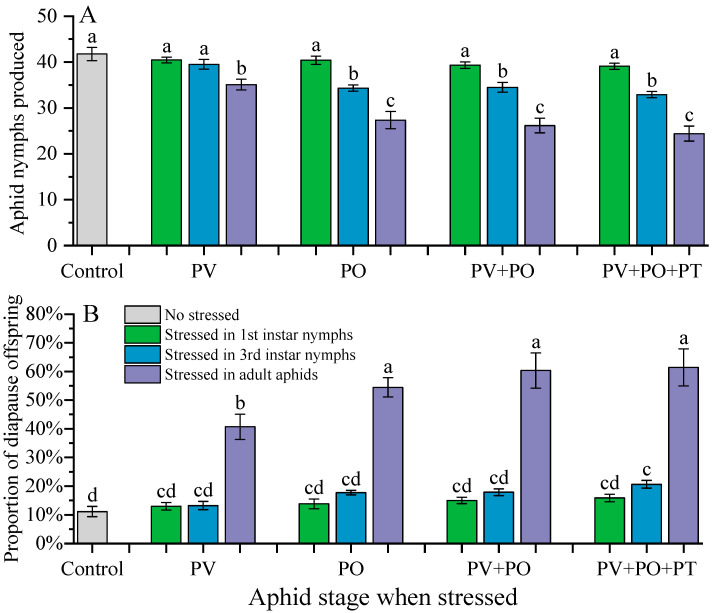
Fecundity (**A**) and proportion of diapause offspring (**B**) of *Periphyllus koelreuteriae* after exposure to predator cues. Different letters above the bars indicate significant differences between different treatments (*p* < 0.05), based on the result of Tukey’s HSD. PV: predator visual cues; PO: predator odor cues; PV + PO: predator visual and odor cues; PV + PO + PT: predator visual, odor, and tactile cues.

The fecundity and proportion of diapause offspring in *P. koelreuteriae* varied depending on the stage of stressed aphids and stress type (Table 4, Figure 2). Overall, the more mature the aphids were, the less they reproduced, but the greater the proportion of diapause offspring. Regardless of the type of stress, reproduction and the proportion of diapause offspring during adult aphid stress were significantly different from those in the nymph-stress group and the control group (Figure 2).

Regardless of which predation risk cues the first-instar nymphs were exposed to, their fecundity and the proportion of diapause offspring were not significantly different from that of the control group (Figure 2).

Compared with the control group, the predator visual cues showed no difference in the reproduction of the third-instar nymphs; however, the other three predation risk cues significantly reduced reproduction (F_4,85_ = 13.844, *p* < 0.001). Additionally, there was no significant difference among these three groups (Figure 2A). Similarly, there was no significant difference in the proportion of diapause offspring between the PV group and the control group. However, the diapause ratio of offspring in the other predation risk groups significantly increased (F_4,85_ = 8.158, *p* < 0.001), and there was no significant difference among the three groups (Figure 2B).

When adult aphids were exposed to different predation cues, all four predation risk treatment groups of ladybug beetles significantly reduced their fecundity (F_4,85_ = 21.868, *p* < 0.001). There was no significant difference between the PO, PV + PO, and PV + PO + PT groups, but all were significantly lower than the PV group (Figure 2A). Similarly, all four predation risk treatment groups of lady beetles significantly increased the diapause ratio of offspring (F_4,85_ = 19.364, *p* < 0.001). The PV + PO and PV + PO + PT groups were significantly higher than the PV group, but not significantly different from the PO group (Figure 2B).

## 4. Discussion

This study indicates that the non-predatory effects of *H. axyridis* on *P. koelreuteriae* are related not only to different predation risk cues from lady beetles, but also to the life stages of aphids under stress. The strongest stress effects for aphids were observed in response to multiple cues from predators, with the stress induced by predator odor cues being stronger than that caused by predator visual cues. Aphids at different developmental stages responded differently to the predation risk. The more mature the aphids were, the more sensitive they became to this risk. The first- and third-instar nymphs did not sense predator visual cues until they reached adulthood. When exposed to predator cues, the development time of nymphs was prolonged, while the adult lifespan was shortened. Additionally, the fecundity of aphids under the stress of predation risk decreased, while the proportion of diapause offspring increased.

When confronted with a predator threat, the anti-predator strategies of prey usually lead to changes in their growth rates [16]. This study showed that the development time of *P. koelreuteriae* nymphs exposed to predation risk cues was significantly prolonged. The development time of 1st–3rd instar stages was significantly prolonged when 1st instar nymphs were exposed to multiple predator cues; 3rd instar nymphs exhibited developmental delay only when their current developmental stage (3rd instar) was stressed. Studies have shown that the growth rate of insects is not only related to feeding but also to metabolic efficiency. Peach aphids reduce phloem feeding time in the presence of predators [27]. Predation risk can reduce the growth rate of *Lestes sponsa* larvae by decreasing their food digestion efficiency and metabolic rate [28]. However, there are also contrasting results. For example, butterfly larvae (*Pararge aegeria*) allocate more energy resources to growth and development in order to develop rapidly when facing predation risks [29]. This difference may be related to energy allocation strategies.

Studies have shown that under predation risk, prey make trade-offs between responding to predation threats and their lifespan [30,31]. In this study, aphid lifespan was shortened when faced with predation risk. The results are in line with previous findings: as predation pressure increases, the development time of *Tyrophagus putrescentiae* larvae is shortened, while the adult lifespan is also shortened [32]. The lifespan of *Spodoptera litura* was similarly shortened under bat predation risk [33]. Long-term exposure to the predation threat from *Menochilus sexmaculatus* significantly shortened the adult lifespan of cotton aphids (*Aphis gossypii*) [34]. Additionally, the adult lifespan of *Bactrocera dorsalis* exposed to the predation risk from *Hierodula patellifera* was also shortened [35]. These results suggest that adults may reduce the time window of exposure to predators by shortening their lifespan and completing reproduction as soon as possible to ensure the continuation of the population under stressed conditions.

We also showed in this study that *P. koelreuteriae* under predation stress resulted in a reduction in fecundity. Both third-instar nymphs and adults in this study exhibited reduced fecundity under predation pressure. This result is consistent with our initial expectation that investment in offspring would be diminished by the detection of predation risk. Several previous studies have shown that prey experiencing predation risk reduced reproduction. For example, *M. persicae* exhibited a 25% reduction in population size when peach aphids decreased the time spent feeding on phloem in the presence of *H. axyridis* odor cues [27]. Phytophagous mites, such as *T. putrescentiae*, under predation pressure, reduce fecundity and allocate resources to anti-predation strategies [36]. *Sitobion miscanthi* also reduced net fecundity due to the presence of isolated predators [37]. Female *B. tryoni* lay fewer eggs in the presence of predator olfactory cues [38]. However, increased prey reproduction due to predation risk has also been reported. Exposing the potato aphid *Macrosiphum euphorbiae* to *Hippodamia convergens*, which were rendered non-lethal by mouthpart manipulation, resulted in significantly higher numbers of nymphs [39]. Female *B. dorsalis* exposed to predation risk had a reduced developmental time but a higher reproductive rate than the control treatment [35]. When threatened by long-term predation, the lifespan of the aphid *Rhopalosiphum padi* was shortened, but the reproduction rate increased [40]. The negative correlation between fecundity and life span indicates that there are trade-offs between reproduction and life span.

There is growing evidence that maternal exposure to predation risk affects the phenotype of prey offspring [40]. Prey mothers exposed to predation risk sometimes alter the morphology and/or anti-predator behaviors of their offspring to reduce predation risk. In the presence of predator risk, pea aphids (*Acyrthosiphon pisum*) and peach aphids (*M. persicae*) increase the production of winged aphid offspring [26,41]. The reproductive capacity of this morphotype is lower than that of the wingless offspring. It is mainly responsible for long-distance transmission and enhances the ability of offspring to evade predators [42]. In this study, compared with the control treatment, aphids exposed to predation risk increased the proportion of diapause offspring. Diapause aphids differed significantly from normal aphids in appearance and behavior, with diapause aphids having transparent bodies and being sedentary (neither eating nor moving) at the edges of leaves, while normal aphids had black speckles on their bodies and were active feeders in the centers of leaves. Compared with normal aphids, diapause aphids have a lower probability of being preyed upon by predators. In fact, caterpillars are more vulnerable to predation when actively feeding than when stationary [43]. The increase in the proportion of diapause offspring suggests transgenerational plasticity regulation. Although it is unclear how the condition information about conditions experienced by mothers is transmitted to offspring, several studies have focused on epigenetic mechanisms to explain this phenomenon [44,45]. Future studies using molecular approaches may reveal the mechanisms by which predators exert transgenerational effects on the morphology and behavior of aphid offspring.

This study confirmed the dominant role of chemical cues in risk perception. The stress effect of odor cues alone was significantly stronger than that of visual cues, which is consistent with the conclusions of most aphid studies. Volatile and contact chemical cues play a decisive role in whether aphids choose to settle on plants [46]. Lady beetle tracks left on leaves cause aphid avoidance and reduce host plant colonization by aphids through contact and olfactory cues, or a combination of both [47]. Vision plays a central role in predator detection in most animals active in light environments, even those with simple visual systems [48]. Several studies have shown that aphids use vision to detect approaching predators. Behavioral responses, such as twitching, kicking, crouching, and dropping from the plant, have been observed in studies testing aphid responses to artificial visual stimuli or passing shadows [49,50,51]. However, it has recently been found that visual cues (hand-held objects and expanding shapes on the screen) can only elicit aphid antennal movements, but not defense responses [52]. Due to their low visual acuity, aphids may struggle to reliably recognize visual predators [53]. Visual cues may be the least important way for aphids to detect the approach of a predator. Notably, visual cues, when acting alone, affected only adult aphids in this study, suggesting that the eyes of aphids may not be fully developed during their immature stages.

Although detecting moments of predator contact relies on the sense of touch, aphids detecting distant approaching predators must rely on the perception of visual, chemical (such as alarm pheromones), and plant-borne vibrational cues. Pea aphids can perceive both predators and non-predators through vibrational and visual cues, utilizing multimodal cues to detect approaching seven-spotted lady beetle predators [52]. The major breakthrough of this study is the revelation of the synergistic effect of multimodal signals, with the combination of odor, visual, and touch cues having the strongest effect. The mechanism by which aphids detect predators may involve a complex interaction among multiple sensory cues. We cannot accurately determine the integration of information from multiple sensory modalities by aphids. This is because it is inherently challenging to design behavioral assays that unravel how animals assess risk via the integration of multisensory information [54]. Animals may also adjust their responses according to their internal state [55,56], and the absence of a response does not necessarily mean that the animal has failed to detect a certain cue [54].

This study revealed a significant developmental-stage dependence of predation risk cues on the regulation of development time in *P. koelreuteriae*. The 1st-instar nymphs only prolonged their developmental time in the early stages (1st–3rd instars) when exposed to multiple predator cues. Third-instar nymphs showed a developmental delay only when their current developmental stage (third instar) was stressed, while fourth-instar nymphs and adults were not significantly affected. This may be related to differences in the sensitivity of developmental insect stages and age-specific energy allocation strategies, but the specific reasons need further investigation. Compared with nymph aphids, adult aphids exhibited a comprehensive stress response. Regardless of the type of exposure to predation risk (including visual cues), the lifespan, reproductive output, and proportion of diapause offspring during adult aphid stress were significantly different from those in the nymph-stress group and the control group. The increased sensitivity of adult aphids to risk cues may reflect evolutionary adaptations. According to the dynamic energy budget theory [57], organisms partition assimilated energy into three areas: maintenance, growth, and reproduction. Under predation stress, adult aphids (which have completed growth investment) prioritize anti-predator resilience over reproductive output. Specifically, energy normally allocated to reproduction is redirected to behavioral vigilance costs (increased scanning activity during feeding). Crucially, diapause offspring require higher per-offspring energy investment than non-diapause morphs due to cryoprotectant accumulation (e.g., lipids, glycerol, and antifreeze proteins) [58]. This explains the observed trade-off: total offspring production decreases because each diapause-destined embryo demands greater maternal resources. Notably, the main limitations of this study include the failure to quantify the stress intensity of different cues and the inability to detect transgenerational effects. It is recommended that follow-up studies combine behavioral ecology and epigenetic approaches to explore the dose–response relationships and transgenerational memory mechanisms of risk cues. Additionally, one caveat of the current study is that it was conducted in small arenas, resulting in highly concentrated cues and limited space, which may not accurately represent this system in nature.

## 5. Conclusions

In summary, this study indicates that the predation risk on *P. koelreuteriae* is related not only to different predation risk cues from lady beetles but also to the life stages of aphids under stress. Nymphs cannot recognize visual cues from predators but can perceive odor cues, while adult aphids could detect the visual, odor, and tactile cues of lady beetles. Nymph aphids sensing lady beetle cues only delayed their current developmental stage and did not affect later development or adult longevity, but they exhibited reduced fecundity. Adult aphids at risk of predation, on the other hand, had shorter lifespans, a lower reproductive output, and an increased proportion of diapause offspring. This study systematically reveals the multilevel regulation of aphid life history strategies by the non-consumption effects of predation risk, providing new insights into the ecological regulation of pests. For example, artificial risk cues were released during the sensitive developmental stage (adult aphid stage) for biological control.

## Figures and Tables

**Table 1:** Developmental time of each insect stage when 1st-instar nymphs were exposed to different predator cues.

Predator Risk	N1	N2	N3	N4	Total Nymph Stage	Adult Longevity
CK	3.14 ± 0.13 c	2.05 ± 0.09 c	1.82 ± 0.09 bc	1.85 ± 0.09 a	8.84 ± 0.20 c	9.72 ± 0.15 a
PV	3.20 ± 0.11 c	2.09 ± 0.08 c	1.87 ± 0.09 bc	1.86 ± 0.09 a	9.03 ± 0.24 c	9.34 ± 0.21 a
PO	3.64 ± 0.15 b	2.39 ± 0.09 b	2.11 ± 0.07 ab	1.95 ± 0.09 a	10.09 ± 0.23 b	9.95 ± 0.34 a
PV + PO	3.65 ± 0.12 b	2.43 ± 0.10 ab	2.14 ± 0.08 ab	2.02 ± 0.11 a	10.25 ± 0.20 b	9.33 ± 0.37 a
PV + PO + PT	4.66 ± 0.16 a	2.66 ± 0.09 a	2.24 ± 0.08 a	2.09 ± 0.12 a	11.65 ± 0.26 a	8.86 ± 0.35 a

CK: control; PV: predator visual cues; PO: predator odor cues; PV + PO: predator visual and odor cues; PV + PO + PT: predator visual, odor, and tactile cues; N1: 1st-instar nymph; N2: 2nd-instar nymph; N3: 3rd-instar nymph; N4: 4th-instar nymph. Different letters within the same column indicate significant differences between the treatments (*p* < 0.05), based on the result of Tukey’s HSD.

**Table 2:** Developmental time of each insect stage when 3rd-instar nymphs were exposed to different predator cues.

Predator Risk	N3	N4	Adult Longevity
CK	1.89 ± 0.10 c	1.83 ± 0.11 a	9.75 ± 0.18 a
PV	2.00 ± 0.09 bc	1.86 ± 0.11 a	9.03 ± 0.20 a
PO	2.31 ± 0.08 ab	1.92 ± 0.07 a	9.08 ± 0.27 a
PV + PO	2.33 ± 0.11 ab	1.94 ± 0.09 a	9.47 ± 0.31 a
PV + PO + PT	2.56 ± 0.11 a	1.94 ± 0.08 a	9.14 ± 0.28 a

CK: control; PV: predator visual cues; PO: predator odor cues; PV + PO: predator visual and odor cues; PV + PO + PT: predator visual, odor, and tactile cues; N3: 3rd-instar nymph; N4: 4th-instar nymph. Different letters within the same column indicate significant differences between the treatments (*p* < 0.05), based on the result of Tukey’s HSD.

**Table 3:** The influence of adult aphids exposed to different predator cues on their lifespan.

Predator Risk	Adult Longevity
CK	9.75 ± 0.18 a
PV	8.78 ± 0.31 b
PO	8.25 ± 0.22 bc
PV + PO	7.61 ± 0.23 cd
PV + PO + PT	7.22 ± 0.29 d

CK: control; PV: predator visual cues; PO: predator odor cues; PV + PO: predator visual and odor cues; PV + PO + PT: predator visual, odor, and tactile cues. Different letters within the same column indicate significant differences between the treatments (*p* < 0.05), based on the result of Tukey’s HSD.

**Table 4:** Two-way ANOVA for the effects of different treatments on fecundity and proportion of diapause offspring in *Periphyllus koelreuteriae*.

Trait	Source	df	Mean-Square Value (MS)	F-Values	*p*
Fecundity	Stressed aphid stage	2	1625.215	104.908	**<0.001**
	Stress type	3	259.581	16.756	**<0.001**
	Stressed aphid stage × Stress type	6	55.928	3.610	**0.002**
	Error	132	15.492		
Proportion of diapause offspring	Stressed aphid stage	2	2.367	191.616	**<0.001**
	Stress type	3	0.077	6.238	**0.001**
	Stressed aphid stage × Stress type	6	0.023	1.845	0.095
	Error	132	0.012		

“×” is an interactive token. Significant values (*p* < 0.05) are printed in bold.

## Data Availability

The original contributions presented in this study are included in the article. Further inquiries can be directed to the corresponding author.
